# Patient perspectives on continuity of care: adaption and preliminary psychometric assessment of a Norwegian version of the Nijmegen Continuity Questionnaire (NCQ-N)

**DOI:** 10.1186/s12913-017-2706-1

**Published:** 2017-11-21

**Authors:** Øystein Hetlevik, Merethe Hustoft, Annemarie Uijen, Jörg Aßmus, Sturla Gjesdal

**Affiliations:** 1 Department of Global Health and Primary Health Care, University of Bergen, PO-box 7804, N-5020 Bergen, Norway; 2 Centre for Habilitation and Rehabilitation in Western Norway, Bergen Local Health Authority, Bergen, Norway; 3 Department of Primary and Community Care, Radboud University Nijmegen Medical Centre, Nijmegen, The Netherlands; 4 Centre for Clinical Research, Haukeland University Hospital, Bergen, Norway

**Keywords:** Continuity of care, Patient reported outcome measure, Healthcare, General practice, Health service research

## Abstract

**Background:**

Continuity of care is regarded as a core quality element in healthcare. Continuity can be related to one or more specific caregivers but also applies to collaboration within a team or across boundaries of healthcare. Measuring continuity is important to identify problems and evaluate quality improvement of interventions.

This study aimed to assess the feasibility and psychometric properties of a Norwegian version of the Nijmegen Continuity Questionnaire (NCQ).

**Methods:**

The NCQ was developed in The Netherlands. It measures patients’ experienced continuity of care across multiple care settings and as a multidimensional concept regardless of morbidity. The NCQ comprises 28 items categorised into three subscales; two personal continuity scales, “care giver knows me” and “shows commitment”, asked regarding the patient’s general practitioner (GP) and the most important specialist; and one “team/cross boundary continuity” scale, asked regarding primary care, specialised care and cooperation between GP and specialist, with a total of seven factors. The NCQ was translated and adapted to Norwegian (NCQ-N) and distributed among patients referred to somatic rehabilitation (*N* = 984, response rate 34.5%). Confirmatory factor analyses (CFA), Cronbach’s alpha, intra-class correlation (ICC) and Bland–Altman plots were used to assess psychometric properties.

**Results:**

All patients (*N* = 375) who had responded to all parts of the NCQ-N were included in CFA. The CFA fit indices (CFI 0.941, RMSEA 0.064 (95% CI 0.059–0.070), SRMR 0.041) support a seven-factor structure in the NCQ-N based on the three subscales of the original NCQ. Cronbach’s alpha showed internal consistency (0.84–0.97), and was highest for the team/cross-boundary subscales. The NCQ-N showed overall high reliability with ICC 0.84–91 for personal continuity factors and 0.67–0.91 for team factors, with the lowest score for team continuity within primary care.

**Conclusions:**

Psychometric assessment of the NCQ-N supports that this instrument, based on the three subscales of the original Dutch NCQ, captures the concept of “continuity of care” among adult patients with a variety of longstanding medical conditions who use healthcare on a regular basis. However, its usefulness among varied patient groups, including younger people, patients with acute disorders and individuals with mental health problems, should be further evaluated.

**Electronic supplementary material:**

The online version of this article (10.1186/s12913-017-2706-1) contains supplementary material, which is available to authorized users.

## Background

Continuity of care is considered to be fundamental to quality healthcare [1, 2]. It is associated with improved patient satisfaction, especially among patients with chronic conditions [1–3], reduced costs and decreased hospital admissions [4–6]. Thus, improving continuity is key to health professionals and other stakeholders providing higher healthcare quality [7]. Continuity of care is a multi-faceted concept [8] that has broadened in recent decades from a focus on personal continuity related to one caregiver, towards personal, informational and management continuity involving several healthcare professionals [9–11].

In general practice, personal doctoring has been highly valued and considered as a core quality element [12]. However, *personal continuity* and commitment is regarded to be the foundation for building trust and promoting partnerships at all levels of healthcare [13]. Furthermore, patients with serious diseases have been reported to appreciate personal attachment to their caregivers [14]. However, an elevated risk for fragmented care is likely in complex health services with large units and several professional groups [15]. Both cooperation between caregivers in teams *(team continuity*) and information handover during a care process *(cross-boundary continuity*) are considered crucial to ensuring high quality of care [5, 10].

The last decade has seen two major healthcare reforms in Norway, both of which aimed to improve continuity of care. When the general practitioner (GP) system in Norway was reorganised into a list patient system in 2001, one main goal was to achieve personal continuity of care by giving each inhabitant a defined regular GP who was expected to have a coordinating role [16]. In 2010, the Norwegian parliament introduced “cooperation reform”, which shifted responsibility for many patients with serious conditions from hospitals to primary care that was organised by the municipalities. One aim of this reform was to improve cooperation between specialised care and local authorities, stating that “patients’ needs for coordinated services are not sufficiently met” [17]. Similar reforms have been introduced in many countries [18, 19].

Measuring continuity of care is important to identify problems and evaluate interventions aimed at improving continuity of care. Continuity of care has often been assessed according to different measures of contact between the patient and different caregivers during a defined period [20, 21]. However, there are arguments for evaluating continuity of care by examining patients’ experiences of provided care as the preferred perspective [21–23]. Several disease-specific, patient-reported tools have been developed to assess patients’ experiences of continuity of care, but few can be generalised across different patient groups [21, 24].

The Nijmegen Continuity Questionnaire (NCQ) measures patients’ experienced continuity of care as a multidimensional concept (personal, team and cross-boundary continuity) regardless of morbidity and across multiple care settings [25]. Furthermore, the NCQ has been confirmed to be a comprehensive, reliable and valid instrument [26].

To use an instrument such as the NCQ in a new language, there is need for linguistic translation and adaption to both the new language and context, a process referred to as cross-cultural adaption. Guidelines are given with recommendations for steps in this process [27].

This study aims to describe the adaptation of the NCQ into a Norwegian version (NCQ-N) and to assess its feasibility and psychometric properties for Norwegian patients with various chronic somatic disorders.

## Methods

Norwegian and Dutch healthcare services are largely similar in that patients are assigned to a regular GP who typically works in a group practice and who has a gate-keeping role regarding use of specialised care. This similarity supports how construct validity assessments performed on the original version of the NCQ are also applicable in Norway, after adaptation to a Norwegian context.

### Nijmegen continuity questionnaire

The instrument comprises 28 items that fall within the following three subscales:

- Personal continuity: care provider knows me (5 items each for the GP and most important specialist)

- Personal continuity: care provider shows commitment (3 items each for the GP and most important specialist)

- Team/cross-boundary continuity (4 items each for collaboration between care providers within general practice, within the hospital/outpatient department and between the GP and specialist).

Items are presented as statements and scored along a 5-point Likert scale ranging from “strongly disagree” to “strongly agree” with the additional option “I do not know”. In the questionnaire, the items are presented in five groups of items, two item groups concerning personal continuity related to the GP and the most important specialist and three item groups with the team/cross boundary scale related to primary care, specialised care and between the GP and specialist. Both the subscales regarding personal continuity are presented as part of the same item group for the GP and specialist, respectively. The participants are instructed to skip the item groups related to the GP and specialist if they had not been in contact in the last 12 months and the other item groups if they were not seen as applicable. Principally, the model has three subscales, but the questions regarding the subscale “personal continuity – healthcare provider knows me” and “personal continuity- healthcare provider shows commitment” are used for both the GP and specialist, and the team/cross boundary subscale is applied to three team contexts: in primary care, in specialised care, and between the GP and specialist, giving a seven-factor model.

### Translation and adaption

The NCQ was translated into Norwegian as recommended in a guideline by Beaton et al. using forward and backward translations based on the original Dutch version with the help of both linguistic and healthcare professionals from both countries [27]. Most general practices in The Netherlands include several healthcare professionals including physiotherapists, counsellors, and health visitors. However, general practices in Norway are usually only serviced by 1–5 GPs who are supported by “secretaries” or nurses. Therefore, for the adaptation to Norwegian context we found it necessary to broaden the scope of the primary care related team continuity items to include cooperation between GPs, nurses, and allied health professionals such as physiotherapists in primary care. Other items remained similar to that of the original, only adapted to the Norwegian language after discussion in the research group and in a pilot test. Based on discussion in the research group and testing the questions in a smaller sample, a direct translation of “very” as used in seven of twelve items was found unnatural in describing a relationship to a professional in the Norwegian language, and was thus left out. In back translation, these items seemed less loaded. We decided to give priority to natural language at the cost of generating a problem with comparison of grading of scales across countries.

The same ratings along a 5-point Likert scale, with an additional option of “I do not know,” were used.

The final version of the NCQ-N was tested on 33 patients in rehabilitation institutions who filled in the whole questionnaire package with research personnel available and were instructed to ask for clarifications. This process revealed no problems in understanding the NCQ-N content.

### Study population and data collection

A cross-sectional survey of patients referred to seven specialised rehabilitation institutions in Western Norway was conducted in the first six months of 2015. Among the 2852 invited to participate, 991 returned the questionnaire, but 7 patients were excluded because they had not given written consent for further use of data. In total, 984 patients were included giving a response rate of 34.5%. Nearly half of patients got a postal invitation at referral and returned the questionnaires by post before the rehabilitation stay and the others were invited when arriving the rehabilitation institution and filled out the questionnaire when starting the stay. Response rates were similar with both ways of recruitment.

The NCQ-N was distributed to the patients as part of a package of validated survey instruments to measure health, quality of care, function and participation in society. We also included questions about health problems and use of healthcare during the previous 12 months. The health problems of the patients were described by combining reason for referral given by GP or specialist, the diagnoses used by rehabilitation institutions and diseases over the last 12 months as reported by the patients and grouped into musculoskeletal, cardiovascular, respiratory, neurological, cancer, endocrinology, urinary tract and mental health problems (Table 1). Comorbidities are described by summing up the number of these groups of diseases for each patient.

**Table 1:** Characteristics of the study population recruited in a survey among patients referred to somatic rehabilitation in Western Norway

	Study population % (*N* = 984)	Non-responders (%) (*N* = 1868)	*P* value
Patient gender			
Female	63.4	67.2	0.043^*^
Male	36.6	32.8	
Age (mean(SD))	58.1 (14.1)	55.6 (16.7)	0.001^**^
Proportions in age groups			
< 40 years	10.2	17.2	<0.001^*^
40–49 years	17.6	21.0	
50–59 years	24.7	22.5	
60–69 years	25.4	15.8	
≥ 70 years	22.1	23.5	
Reported diseases, grouped			
Musculoskeletal	70.9		
Cardiovascular	35.5		
Respiratory	23.3		
Neurology	13.8		
Cancer	15.4		
Endocrinology	16.5		
Skin	21.4		
Urinary tract	4.6		
Mental health	23.9		
Sum reported diseases, according to list above			
1	30.0		
2	35.2		
3 or more	34.8		
Referred by			
General practitioner	61.6		
Specialists	27.9		
Others / missing	10.5		
Use of health service (≥1 contacts last 12 months):			
GP	97.3		
Specialist, hospital	64.4		
Specialist, private	35.7		
Specialist total (hospital and/or private)	75.6		
Psychologist	12.6		
Physiotherapist	60.6		
Occupational therapist	10.7		
Home care contact	10.7		
Hospitalized	48.4		
Stay in rehabilitation institution	22.6		
Stay in a nursing home	2.3		

^*^Pearson chi square. ^**^Student T-test

Data from all included participants were assessed to examine response patterns of the different NCQ-N items and subscales and to analyse for internal consistency using Cronbach’s alpha.

Because the participants were instructed to skip an item group if the items were not seen as applicable, e.g., questions about the specialists for patients who have not seen a specialist, the data contained entire item groups left empty. Assuming that completely empty item groups indicate that the questions were not applicable, we used two grades of restrictions. First, we selected all patients with responses, including “I do not know”, in all item groups (*N* = 375). Then, for sensitivity analyses we also selected a sample with responses to the item groups about GPs and specialists, but not necessarily the team/cross-boundary items (*N* = 652).

Test of reproducibility (test-retest) was based on a randomly selected sample of participants (*N* = 116) who answered all survey questions again two weeks after their first response.

### Statistical analyses

A subscale was calculated as the mean of all items in the subscale, excluding cases with more than one item missing. In analyses we treated “I do not know” as a missing.

Psychometric properties of the NCQ-N were assessed using confirmatory factor analyses (CFA) with robust maximum likelihood estimation (Yuan–Bentler-correction) and Cronbach’s alpha.

In CFA missing was handled by Full Information Maximum Likelihood (FIML). Using this method we found it most correct to include only respondents who had responded to all item groups (*N* = 375), and not estimate values for patients who had not found the item group applicable. However, for sensitivity analyses we also used a less restrictive sample of 652, described above.

In CFA, the following fit indices were calculated, with values indicating a good fit within parentheses: root mean square error of approximation (RMSEA) (<0.05 indicates a good fit and up to 0.08 mediocre fit) [28], comparative fit index (CFI) (> 0.9) [29], standardized root mean square residual (SRMR) (<0.08) [30], the relative/normed chi-square (X^2^/df < 2 good, < 5 acceptable) and chi-square test (*p* > 0.05) [29, 31, 32].

Cronbach’s alpha was considered satisfactory between 0.70 and 0.95 [33]. We also present the item-rest correlations, to test the correlation between each item in a subscale and the subscale without the actual item.

If more than 15% of the samples have the highest or lowest possible score, referred to as floor or ceiling effect, the instrument’s ability to discriminate between responses is reduced [33]. We therefore estimated the proportion with highest and lowest possible scores in all subscales.

Correlation between subscales was analysed using Pearson product-moment correlation. A correlation coefficient between 0.3 and 0.5 was considered moderate, and >0.5 a strong correlation [34].

Intra-class correlation (ICC) was used in the test-retest analysis using a two-way mixed effect model with absolute agreement, and ICC >0.70 indicates acceptable reproducibility [33].

The statistical program R 3.3 [35] with the package lavaan 0.5 [36] was used to perform the factor analyses; otherwise, Stata Statistical Software (Release 14; College Station, TX, USA) was used. The graphics were created using Matlab 9.0 (The MathWorks Inc., Natick, MA).

## Results

The characteristics of the study population (*N* = 984) are shown in Table 1, compared with some characteristics of the non-responders in the survey.

The responses to different items in the NCQ-N are described in Table 2. In the total sample of 984, only 15 skipped the item group concerning GPs, and “I do not know” was chosen for 0.2%–6.4% in this item group. These measures were markedly higher in the other item groups. The item group related to team continuity within specialised care was skipped by 45% of respondents. The proportion answering “I do not know” was highest regarding team continuity within primary care, with figures slightly above 20%.

**Table 2:** Responses to each items^a^ in the Norwegian version of Nijmegen Continuity Questionnaire (NCQ-N) in a survey among patients referred to somatic rehabilitation in Western Norway (*N* = 984)

Item groups	Responses to Likert scale 1–5^b^				Do not know^b^	Missing^c^	
	n (%)	Mean (SD)	% lowest	% highest	n (%)	All within item group, n (%)	other, n (%)
About GP							
GP1	955 (97.1)	3.99 (0.95)	2.0	33.3	2 (0.2)	15 (1.5)	12 (1.2)
GP1	955 (97.1)	4.02 (0.84)	1.6	40.2	9 (0.9)	15 (1.5)	5 (0.5)
GP3	932 (94.7)	4.19 (0.80)	1.0	37.6	14 (1.4)	15 (1.5)	23 (2.3)
GP4	907 (92.2)	3.58 (1.15)	5.1	24.9	43 (4.4)	15 (1.5)	19 (1.9)
GP5	918 (93.3)	3.63 (1.07)	3.4	21.4	30 (3.1)	15 (1.5)	21 (2.1)
GP6	878 (89.2)	3.36 (1.21)	8.9	19.1	64 (6.5)	15 (1.5)	27 (2.7)
GP7	900 (91.5)	3.78 (0.98)	3.6	24.0	40 (4.1)	15 (1.5)	29 (3.0)
GP8	882 (89.6)	3.41 (1.10)	6.1	17.0	57 (5.8)	15 (1.5)	30 (3.1)
Team primary care							
TPC1	454 (46.1)	2.92 (1.00)	9.5	4.9	209 (21.2)	316 (32.1)	5 (0.5)
TPC2	428 (43.5)	2.91 (0.92)	7.7	4.2	230 (23.4)	316 (32.1)	10 (1.0)
TPC3	456 (46.3)	2.95 (0.99)	8.8	4.6	200 (20.3)	316 (32.1)	12 (1.2)
TPC4	438 (44.5)	2.76 (0.99)	11.6	3.9	220 (22.4)	316 (32.1)	10 (1.0)
About specialist							
SP1	617 (62.7)	2.94 (1.18)	13.0	10.2	32 (3.3)	332 (33.7)	3 (0.3)
SP2	586 (59.6)	3.48 (1.08)	5.1	16.0	57 (5.8)	332 (33.7)	9 (0.9)
SP3	556 (56.5)	3.53 (1.05)	4.7	16.9	79 (8.0)	332 (33.7)	17 (1.7)
SP4	563 (57.2)	2.57 (1.17)	20.3	7.5	75 (7.6)	332 (33.7)	14 (1.4)
SP5	574 (58.3)	2.71 (1.18)	17.4	7.7	62 (6.3)	332 (33.7)	16 (1.6)
SP6	535 (54.4)	2.95 (1.14)	17.4	7.9	105 (10.7)	332 (33.7)	12 (1.2)
SP7	558 (56.7)	3.31 (1.11)	9.9	13.3	83 (8.4)	332 (33.7)	11 (1.1)
SP8	534 (54.3)	2.86 (1.10)	17.4	8.1	101 (10.3)	332 (33.7)	17 (1.7)
Team specialised care							
TSP1	447 (45.4)	3.31 (1.03)	6.9	8.8	92 (9.4)	443 (45.0)	2 (0.2)
TSP2	430 (43.7)	3.34 (0.99)	5.4	8.8	106 (10.8)	443 (45.0)	5 (0.5)
TSP3	438 (44.5)	3.28 (1.01)	6.6	8.5	96 (9.8)	443 (45.0)	7 (0.7)
TSP4	426 (43.3)	3.14 (1.06)	8.9	8.5	112 (11.4)	443 (45.0)	3 (0.3)
Between GP and specialist							
TB1	534 (54.3)	3.43 (0.98)	5.2	9.9	136 (13.8)	306 (31.1)	8 (0.8)
TB2	502 (51.0)	3.27 (0.96)	5.2	8.8	161 (16.4)	306 (31.1)	15 (1.5)
TB3	524 (53.3)	3.37 (0.98)	5.5	9.4	139 (14.1)	306 (31.1)	15 (1.5)
TB4	504 (51.2)	3.21 (1.00)	6.6	8.3	163 (16.6)	306 (31.1)	11 (1.1)

^a^) The content of items are shown in Table 3

^b^) Mean score based in Likert scale 1–5: 1 = strongly disagree, 2 = disagree, 3 = neutral, 4 = agree, 5 = strongly agree with an additional option to answer, “I do not know”

^c^) Missing on all item within an item group is interpreted as a response to the instruction to skip the item group when seen as not applicable by the respondent

There was a high proportion of maximum scores for the items regarding continuity with the GP, indicating a ceiling effect on item level, highest for the item referring to “knowing my medical history well”, where 40% strongly agreed (for item contents, see Table 3). Regarding specialists, there was a floor effect, with 17%-20% of respondents using the lowest score in four of the eight items.

**Table 3:** Subscales scores and measures of internal consistency within subscales in a Norwegian version of Nijmegen Continuity Questionnaire used among patients referred to somatic rehabilitation

Personal continuity - care provider knows me	GP	Specialist	
Subscale items:	*IRC* ^*a*^	*IRC* ^*a*^	
* I know this care provider well*	*0.78*	*0.81*	
* This care provider knows my medical history well*	*0.80*	*0.82*	
* This care provider always remembers what he/she did during my last visit(s)*	*0.76*	*0.75*	
* This care provider knows my family circumstances well*	*0.75*	*0.77*	
* This care provider knows well what I do in my day-to-day life*	*0.72*	*0.80*	
Total subscale score (SD)	3.92 (0.82)	3.05 (1.00)	
Patients with lowest subscale score (floor effect), n (%)	0.7	3.6	
Patients with highest subscale score (ceiling effect), n (%)	14.7	5.2	
Internal consistency (Cronbach alpha)	0.91	0.92	
Personal continuity - care provider shows commitment	GP	Specialist	
Subscale items:	*IRC* ^*a*^	*IRC* ^*a*^	
* This care provider contacts me when necessary without me having to ask him/her to do so*	*0.68*	*0.75*	
* This care provider knows well what I think is important when it comes to my care*	*0.68*	*0.76*	
* This care provider maintains enough contact with me when I am seen by other care providers*	*0.73*	*0.79*	
Total subscale score (SD)	3.51 (0.96)	3.05 (1.0)	
Patients with lowest subscale score (floor effect), n (%)	3.1	9.1	
Patients with highest subscale score (ceiling effect), n (%)	11.8	5.4	
Internal consistency (Cronbach alpha)	0.84	0.88	
Team/cross-boundary continuity -	within primary care	within specialized care	between GP and specialist
Subscale items:	*IRC* ^*a*^	*IRC* ^*a*^	*IRC* ^*a*^
* These care providers pass on information to each other well*	*0.97*	*0.92*	*0.86*
* These care providers work together well*	*0.89*	*0.94*	*0.90*
* The care given by these care providers is well-connected*	*0.89*	*0.91*	*0.85*
* These care providers always know well what the other care providers have done*	*0.88*	*0.88*	*0.84*
Total subscale score (SD)	2.88 (0.91)	3.26 (0.98)	3.33(0.91)
Patients with lowest subscale score (floor effect), n (%)	5.4	5.1	3.6
Patients with highest subscale score (ceiling effect), n (%)	2.8	6.7	6.8
Internal consistency (Cronbach alpha)	0.96	0.97	0.95

^a^Item rest correlation (IRC): Correlation between the actual single item and the subscale without this single item

### Factor structure

The fit indices found in the two different subsamples were rather similar but best in the least strict model (*N* = 625). However, in this sample the proportion of missing, including “I do not know”, was up to 50% in some items. Therefore, we chose to present data for the strictest sample (*N* = 375) with 19%–30% missing, including “I do not know”, in the team factors; 8%–16% missing items in all factors related to specialist; and less than 6% in items regarding GPs. This subsample had a higher proportion of male responders (41.9%) than the total study sample (Table 1), and mean age was 57.2 years, compared with 58.1. In this subsample, three or more diseases were more often reported (42.1% compared with 34.8%), but musculoskeletal problems were less frequent (66.9% compared with 70.9%).

**Fig. 1:**
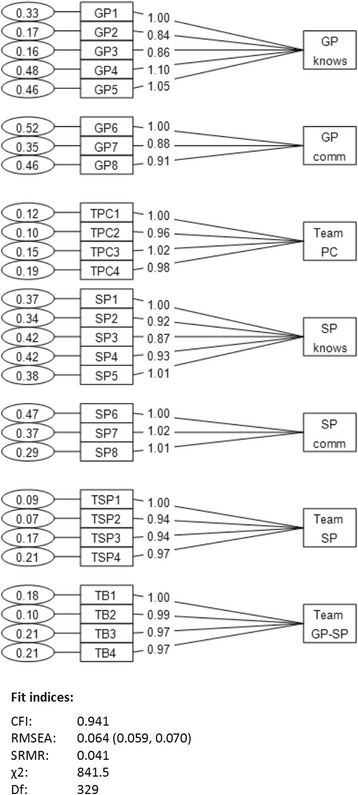
The factor structure found by confirmatory factor analyses in the Norwegian version of the Nijmegen Continuity Questionnaire. Factors: Personal continuity - GP/specialist knows me (GP/SP knows), and Personal continuity - GP/specialist shows commitment (GP/SP comm), Team continuity within primary care (Team PC) and specialised care (Team SP), and Cross-boundary continuity between GP and specialist (Team GP-SP)

The 7-factor model according to the original NCQ fitted the data best [26] (see Fig. 1), compared with a model treating personal continuity as one factor and a two-layer model (see Additional file 1). The factor loadings showed relatively similar values for most items in all seven latent factors. The fit indices for this 7-factor model showed: CFI 0.941, RMSEA 0.064 (95% CI 0.059–0.070), SRMR 0.041, and chi-square 841.5 (*p* < 0.05) with 329 degrees of freedom giving X^2^/df = 2.6.

### NCQ-N single items and subscales

The item group in relation to personal continuity was divided into two subscales, in line with the original NCQ [26], and as supported by the present CFA (Fig. 1). The subscales scores are shown in Table 3, where the items constituting the scales are also listed. Cronbach’s alpha scores were between 0.84 and 0.97, with highest internal consistency found for the team/cross boundary scales (Table 3).

In the two subscales measuring personal continuity for GPs, 14.7% and 11.8% had maximal scores, while the scores for the other subscales ranged from 2.2% to 6.1%. The lowest possible score was seen in between 0.7% and 9.1% in the different sub scales.

A strong correlation was found between the “knows me” and “shows commitment” subscales for GPs and specialists, respectively; however, a low to moderate correlation was identified between these subscales for GPs and specialists (Table 4). Moderate to strong correlations were found between all personal continuity scores and the cross-boundary continuity with correlation coefficients between 0.41 and 0.60.

**Table 4:** Correlation between the subscale scores in the Norwegian version of the Nijmegen Continuity Questionnaire

		Personal continuity				Team/cross boundary continuity		
	N	GP knows me	GP shows commitment	Specialist knows me	Specialist shows commitment	Within primary care	Within specialised care	Between GP and specialist
Personal continuity								
GP knows me	923	1.00						
GP shows commitment	900	0.73**	1.00					
Specialist knows me	563	0.25 **	0.26**	1.00				
Specialists shows commitment	552	0.22**	0.34**	0.82**	1.00			
Team/cross boundary continuity								
Within primary care	426	0.31**	0.41**	0.32**	0.41**	1.00		
Within specialised care	432	0.07	0.15**	0.39**	0.41**	0.43**	1.00	
Between GP and specialist	499	0.46**	0.60**	0.41**	0.50**	0.52**	0.50**	1.00

*p < 0.05; **p < 0.01

### Test-retest

**Fig. 2:**
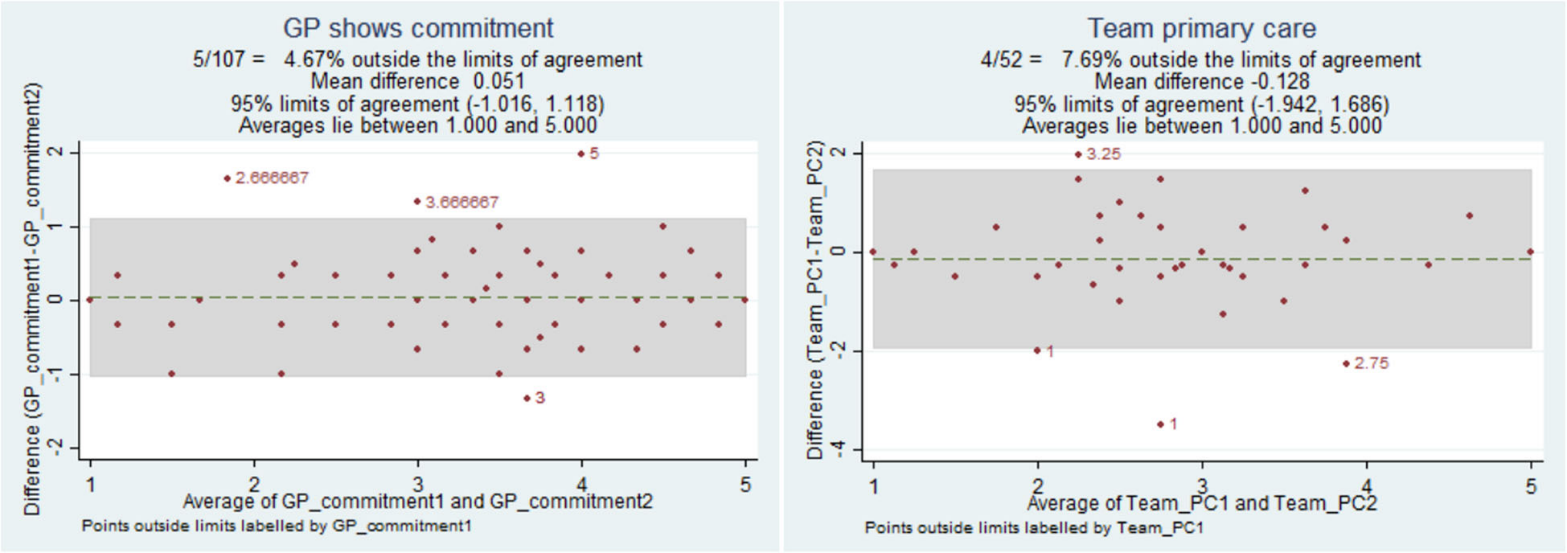
Bland–Altman plots to test reliability, exemplified by the subscales for “Personal continuity –GP shows commitment” and team continuity in primary care

Test-retest reliability was assessed using ICC in a separate sample from the main survey (Table 5). The scores concerning team continuity in primary care had the lowest reliability with an ICC of 0.67, while other factors had an ICC in the range of 0.81–0.91. In the Bland–Altman plots, the mean difference in the scores for continuity in primary care was in line with that of other subscales, but the range of the 95% limits of agreement was larger compared with that of other subscales. The Bland–Altman plots of the subscales with highest and lowest ICC, “personal continuity– GP shows commitment” and team continuity in primary care, are presented as illustrations in Fig. 2.

**Table 5:** Reliability of the subscales in the Norwegian version of the Nijmegen Continuity Questionnaire

		Intra-class correlation		Bland-Altman plot	
	N	Coefficient	95% CI	Mean difference	95% limits of agreement
Personal continuity					
- GP knows me	112	0.89	0.84 to 0.93	0.126	−0.877 to 1.130
- GP shows commitment	107	0.91	0.87 to 0.94	0.051	−1.016 to 1.118
- Specialist knows me	58	0.84	0.72 to 0.91	−0.238	−1.673 to 1.197
- Specialist shows commitment	54	0.88	0.79 to 0.93	−0.148	−1.482 to 1.185
Team/cross boundary continuity					
- Within primary care	52	0.67	0.42 to 0.81	−0.128	−1.942 to 1.686
- Within specialised care	38	0.81	0.64 to 0.90	−0.228	−1.557 to 1.101
- Between GP and specialist	48	0.91	0.84 to 0.95	−0.106	−1.209 to 0.997

Based on a sample (*N* = 116) of participant in a study among patients referred to somatic rehabilitation in Western Norway

## Discussion

### Main findings

Psychometric assessment based on test-retest, Cronbach’s alpha and CFA indicated that the three subscales from the original NCQ can measure continuity of care regarding different caregivers and team contexts among patients with chronic conditions in a Norwegian setting.

### NCQ-N assessment

Our findings show that a seven-factor model could be used to report patient perspectives of continuity in healthcare as a summary of the underlying single items constituting the latent factors. Fit indices were overall acceptable or good; however, a significant chi-squared test indicated possible problems with the model, which could be explained by the high correlation found between subscales.

Cronbach’s alpha was above that recommended for subscales concerning team/cross-boundary continuity. Additionally, the item rest correlations were high on these subscales, indicating a possible overlap in content between single items [33].

There is a marked ceiling effect on single items constituting the personal continuity scales regarding GPs. However, the “GP knows me” scale showed a proportion of maximal scores (14.7%) that is slightly below to the recommended maximum of 15% for a well-designed instrument [33]. The subscale “GP shows commitment” also had a high proportion with maximal scores (11.8%). These findings may reflect the change of loading by removing “very” in the adaptation process. However, regarding the specialist, the single items in subscales showed a floor effect with equally worded items asked for GPs, so the effect of phrasing the items in the adaptation process is not quite clear. In subscales, this adaptation to the Norwegian language appeared to result in acceptable scale properties. On the other hand, a direct comparison of scale scores with the original Dutch version should not be done since the “loading” of some items is changed. However, such comparisons should always be done with precaution since response styles differs between countries and languages [37].

There was very high correlation between the personal continuity scales for GPs and specialists, respectively, but moderate to low correlation comparing GP and specialist scores. This may reflect that patients with more comprehensive care by a specialist have a less close bond with their GP. The cross-boundary continuity scale correlates moderate to high to the all other scales, and might indicate that continuity at different levels is connected in some way. Not surprisingly, there was low correlation between personal continuity related to GPs and team continuity within specialised care. However, reported personal continuity regarding specialists showed moderate to strong correlation to all team settings. This indicates that a patient with need for specialised care also experiences better continuity of care related to different caregivers. This pattern is similar in the Dutch version [26].

Questions regarding team continuity at the primary care level had the lowest ICC, compared with the other factors. The introduction to this item group in the questionnaire was changed in the NCQ-N to include health professionals working in primary healthcare with the same patients as the GPs, as a replacement for team continuity within the Dutch GP practices. However, patients may have experienced difficulty distinguishing between the different care providers, which may explain why this subscale had the lowest reliability compared with all other subscales. Thus, this adaption of the NCQ-N requires further evaluation.

When testing the original version of the NCQ, the questionnaires were distributed directly to the patients by their GPs or their specialists [26]. The patients had at least one chronic condition, and participants were assumed to have had contact with both GPs and hospital specialists. In the present study, questionnaires were distributed by mail or personally at the rehabilitation institution, outside the patients’ usual healthcare settings. Our presumption was that most of these patients had contact with GPs, specialists and other healthcare professionals during the last 12 months because they were referred to a multidisciplinary rehabilitation program. This was apparently not the case, since the majority of missing responses in the NCQ-N were due to skipping item groups in the questionnaire, as the patients were instructed to do in the introduction to each item groups if not seen applicable. However, because some participants seemed to consider groups of items as not applicable despite reporting healthcare contacts that should have given them experience with the actual services, a more instructive introduction to each item group should be considered. The team/cross-boundary scales had 10%–20% with “I do not know” as a response, indicating difficulties in evaluating the informational or management continuity within healthcare.

In future research to test psychometric properties of the NCQ-N, it would have been preferable to include patients with a more uniform use of healthcare. This could be achieved by using the NCQ-N related to more specific care pathways, as COPD, rheumatological or neurological diseases with patient groups using both primary care and specialised care. Such an approach would make all parts of the questionnaire more relevant for most patients.

Despite problems with missing responses in our setting, the present psychometric analyses indicate that the NCQ-N can be a valuable instrument in research and quality development in Norwegian health services. Owing to policy changes, primary care is intended to take greater responsibility for more patient groups [17]. Still, physicians have the main responsibility for medical treatment [16], but more often they do their work in cooperation with other health care professionals at all levels of healthcare [15, 18]. To follow the patient’s experience of personal continuity of care related to GPs and specialist is therefore a useful quality control during changes [1, 13–15, 18]. At the same time, continuity across professional groups also needs assessment from a patient perspective [10, 21, 22]. The structure of NCQ giving the patients instruction to skip not applicable items groups, makes this a generic instrument that can be used across care pathways for different conditions and in different geographical areas. It can also be used to reflect changes over time and between patient groups.

### Study strengths and limitations

One study strength is that the study sample for psychometric analyses consisted of patients with different somatic and mental conditions, and a broad experience with healthcare.

One weakness is low response rate in the survey and our limited information about the non-respondent group. This limits claims of representativeness. The NCQ-N was distributed as a part of a larger battery of questionnaires. This may have decreased motivation to participate in general and partly explain a low response rate in the survey. How to adapt the NCQ to a large population survey setting deserves further study.

Further, only 375 respondents had answered related to all item groups and were included in the CFA. However, this is a sufficient number considering the total of 28 questionnaire items, and is above the recommended limit of 4–10 participants per item [33]. Additionally, the subsample with response to all parts of the NCQ-N, used for validity assessment, was largely similar to the total sample of study participants, but the subsample’s reported higher disease burden less dominated by musculoskeletal problems and a greater use of healthcare were the most marked differences.

One study design limitation is that testing of construct validity was not included. This could have been addressed by comparing the NCQ-N to other instruments that measure continuity of care, validated in Norway, but no such instruments were identified. However, we tested the NCQ-N in healthcare settings similar to those used for validating the original version, and adapted according to recommendations [27]. The construct “continuity of care” is outlined extensively in international literature [10] and we believe that interpretation of this construct is similar across countries with similar social and healthcare systems to The Netherlands and Norway. The comprehensive work that was done when selecting the key dimensions of the NCQ and the testing of construct validity should also apply to the NCQ-N [25].

## Conclusions

Based on a limited number of participants, we chose to present this as a preliminary psychometric assessment of the NCQ-N. However, despite the referred limitations, the NCQ-N seems to be a valid instrument that can be used in future evaluations of healthcare performance and quality assurance in healthcare organisations at different levels of care among adult patients with a variety of longstanding medical conditions and who use healthcare on a regular basis. Using the subscales “Personal continuity – GP/specialist knows me”, “Personal continuity – GP/specialist shows commitment” and “Team/cross-boundary continuity” to assess different contexts of healthcare is considered to capture the concept “continuity of care”. However, the NCQ-N’s usefulness should be further evaluated among other patient groups, including younger people, patients with acute disorders and those with mental health problems.

## Additional file

Additional file 1: Alternative factor models tested for NCQ-N. (TIFF 110 kb)

## Abbreviations

CFA: confirmatory factor analyses
CFI: comparative fit index
FIML: Full Information Maximum Likelihood
GP: General practitioner
ICC: Intra-class correlation
NCQ: Nijmegen Continuity Questionnaire
NCQ-N: Norwegian version of Nijmegen Continuity Questionnaire
RMSEA: root mean square error of approximation
SRMR: standardized root mean square residual
