# Supplementary figures and images for: Patient perspectives on continuity of care: adaption and preliminary psychometric assessment of a Norwegian version of the Nijmegen Continuity Questionnaire (NCQ-N)
Source: BMC Health Serv Res. 2017 Nov 21;17:760. doi: 10.1186/s12913-017-2706-1 (PMC5698962; doi:10.1186/s12913-017-2706-1)

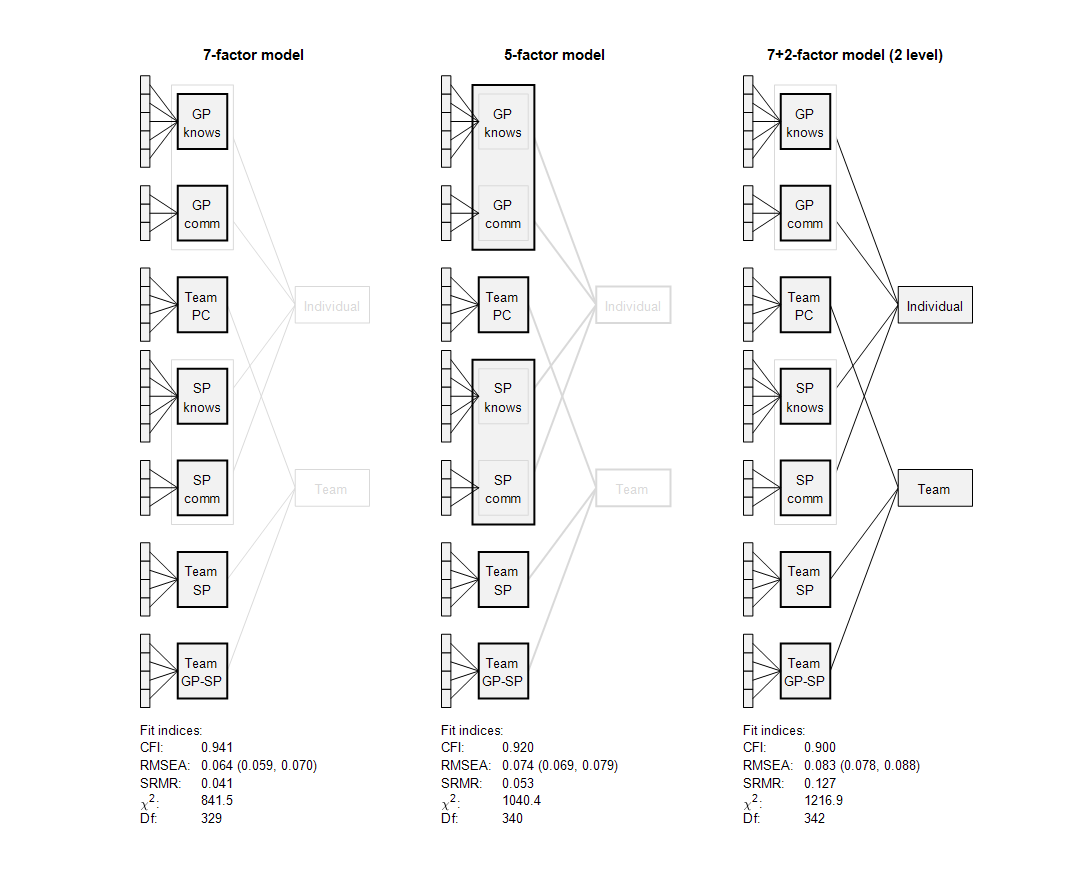

Supplement: Additional file 1: — Alternative factor models tested for NCQ-N. (TIFF 110 kb) [file 12913_2017_2706_MOESM1_ESM.tif]
